# Clustering of protein domains for functional and evolutionary studies

**DOI:** 10.1186/1471-2105-10-335

**Published:** 2009-10-15

**Authors:** Pavle Goldstein, Jurica Zucko, Dušica Vujaklija, Anita Kriško, Daslav Hranueli, Paul F Long, Catherine Etchebest, Bojan Basrak, John Cullum

**Affiliations:** 1 Department of Mathematics, University of Zagreb, Bijenicka 30, 10000 Zagreb, Croatia; 2 Department of Genetics, University of Kaiserslautern, Postfach 3049, 67653 Kaiserslautern, Germany; 3 Department of Molecular Biology, Rudjer Boskovic Institute, Bijenicka 54, 10000 Zagreb, Croatia; 4 Mediterranean Institute for Life Sciences, Mestrovicevo setaliste bb, 21000 Split, Croatia; 5 Faculty of Food Technology and Biotechnology, University of Zagreb, Pierottijeva 6, 10000 Zagreb, Croatia; 6 The School of Pharmacy, University of London, 29/39 Brunswick Square, London, WC1N 1AX, UK; 7 INSERM U- 571, Faculté de Médecine, Université Paris V, 156 rue de Vaugirard, 75730 Paris Cedex 15, France; 8 Equipe de Bioinformatique Génomique et Moléculaire, INSERM U-726, Université Denis Diderot - Paris 7, 2 place Jussieu, 75251 Paris Cedex 05, France

## Abstract

**Background:**

The number of protein family members defined by DNA sequencing is usually much larger than those characterised experimentally. This paper describes a method to divide protein families into subtypes purely on sequence criteria. Comparison with experimental data allows an independent test of the quality of the clustering.

**Results:**

An evolutionary split statistic is calculated for each column in a protein multiple sequence alignment; the statistic has a larger value when a column is better described by an evolutionary model that assumes clustering around two or more amino acids rather than a single amino acid. The user selects columns (typically the top ranked columns) to construct a motif. The motif is used to divide the family into subtypes using a stochastic optimization procedure related to the deterministic annealing EM algorithm (DAEM), which yields a specificity score showing how well each family member is assigned to a subtype. The clustering obtained is not strongly dependent on the number of amino acids chosen for the motif. The robustness of this method was demonstrated using six well characterized protein families: nucleotidyl cyclase, protein kinase, dehydrogenase, two polyketide synthase domains and small heat shock proteins. Phylogenetic trees did not allow accurate clustering for three of the six families.

**Conclusion:**

The method clustered the families into functional subtypes with an accuracy of 90 to 100%. False assignments usually had a low specificity score.

## Background

Rapid progress in DNA sequencing is generating large numbers of deduced protein sequences. The prediction of their function is an important problem in Bioinformatics. This is tackled by comparing new sequences to known sequences as high sequence similarity usually indicates related function. It is possible to use similarity search algorithms such as BLAST [1]. A more sensitive approach is to use hidden Markov models (HMMs) to define protein families as implemented in HMMER suite of programs [2]. Such HMM profiles are used to define protein families in the Pfam database [3]. In many cases, these families consist of functional domains in larger proteins.

In many cases protein families can be split into sub-types based on functional differences e.g. substrate specificity such as for the malonyl-CoA- and methylmalonyl-CoA-incorporating acyl transferase domains of modular polyketide synthetases [4,5]. These differences usually correlate with specific differences in amino acid sequence, which help to understand the molecular basis of protein function and serve as a basis for building prediction programs [6]. In order to identify such diagnostic amino acids, it is first necessary to produce a multiple alignment of the protein sequences to identify corresponding residues in different members of the family. This can be done in various ways e.g. using an HMM-profile [2] or a multiple alignment program such as ClustalW [7]. In some cases, it is possible to identify diagnostic residues merely by inspection of sequences (e.g. [8,9]), but this is difficult or impossible in many cases.

An interesting approach that analysed the entropy associated with different residue positions was described by Hannenhalli and Russell [10]. The biological idea behind this approach is that amino acid residues that are important in the determination of functional subtypes will have different constraints depending on the subtype. In general they will not be absolutely conserved, but evolution will only allow limited variation and the pattern of variation will be different for different subtypes. The functional subtypes corresponding to each protein are input to the program and the program uses an entropy measure to identify residues that split the dataset between the functional subtypes. The detection of specificity-determining residues has been developed further [11-14]. The residues identified by these methods can be used to assign new sequences to the correct subtype. However, it must be emphasized that all these methods rely on experimental data about the subtypes of a sufficiently large collection of proteins to identify the residues.

In many cases of interest there may not be enough experimental data about subtypes, but there is usually a much larger set of protein sequences (deduced from DNA sequences) which have not been experimentally characterised. In this paper we describe a method which divides a set of protein sequences into subtypes based solely on sequence data without any prior assignment of subtypes. The method clustered six well-characterised protein families into functional subtypes without any prior knowledge of protein properties and identified specificity-determining amino acid residues.

## Results and Discussion

### Identification of subtypes

The starting point for the analysis was a multiple sequence alignment of the protein family being analysed. We used ClustalW and ClustalX [7,15] to align sequences [see Additional file 1]. Any other method of generating multiple alignments could be used e.g. with an HMM-profile of the family as implemented in the HMMER suite of programs [2]. The program only considers columns in the multiple alignment which contain amino acids for every member of the protein family (i.e. positions with any gaps are ignored). The program analyses the amino acids present at a given position and performs a statistical test to determine whether the distribution of the amino acids is more compatible with a model that they cluster around a single amino acid or with a model that they cluster around two or more different amino acids; the number of clusters is given to the program as a parameter. The two amino acid model has proved most useful for the six cases considered in this paper i.e. a binary split of the family into two subtypes is attempted. The statistical test needs a model for the substitution of amino acid residues and the BLOSUM-50 matrix [16] was used, which represents the observed substitutions in a large sample of proteins. Although this model will not be strictly true for each amino acid position, the success of the program shows that it is adequate. An evolutionary split statistic was defined (see Methods) that measures how well the position fits the multiple amino acid model i.e. a large value of the statistic indicates that the position should be important in the discrimination between subtypes.

On the basis of the evolutionary split statistic, the user selects a series of positions (a "motif") to be used for splitting the protein family into subtypes. These are typically positions with the best scores, but other criteria (e.g. residues in a particular region or residues close to the active site if a 3-D structure of a family member or a related protein is available) can be used. The clustering algorithm used gives log likelihood values for each sequence that show how well the "motif" assigns the sequence to a particular class. When a division into two subtypes is being carried out, it is useful to use the "specificity score", which is the difference between the log likelihoods for assignment to the two classes. The specificity score is a measure of how good the assignment to the class with higher likelihood is. The user can experiment with different numbers of motif positions to find a selection that gives good discrimination. As we will show later, in most cases this choice is not critical for the success of the method.

### Performance of the program

The program was tested on six different protein families [see Additional file 2]. Nucleotidyl cyclases have two functional subtypes corresponding to use of the substrates ATP or GTP respectively. We extracted 75 sequences (33 adenylate cyclases, 42 guanylate cyclases) from the UniProt database [17]. When the five positions with the best evolutionary split statistic were used to divide the family into two subtypes, the resulting groups were exactly the adenylate and guanylate cyclases (100% accuracy). Five of the ten best positions corresponded to amino acids that were discussed by Hannenhalli and Russell [10] as important in determining the functional subtype (Table 1).

**Table 1:** Nucleotidyl cyclases: residues with best evolutionary split scores.

	**Residue number in multiple alignment**		**Substrate**	
**Evolutionary split score**	**This paper**	**Hannenhalli** **and Russell,** **2000**	**ATP**	**GTP**

113	1509	-	**C**	**V**

110	1636	1020	**W**	**F**

110	1634	1018	**D**	**C**

109	1630	1014	**K**	**M**

91	1517	919	**I**	**Y**

86	1580	-	**F**	**M**

84	1533	935	**E**	**Y**

83	1440	-	**M**	**E**

83	1497	-	**C**	**Y**

81	1656	-	**H**	**Q**

The ten residues with the best evolutionary split scores in the multiple sequence alignment of the nucleotidyl cyclases. When the residue had been detected in previous work [10] the corresponding residue number is given. The dominant amino acid for the two subtypes is shown.

The protein kinase family can be divided into serine/threonine and tyrosine kinases. 215 kinase sequences (85 serine/threonine, 130 tyrosine) were extracted from the protein kinase resource database [18]. When the 7 best positions were used, the program divided the kinases into subtypes with 100% accuracy. Seven of the best ten positions were identified previously as important for the subtype determination [10].

**Figure 1:**
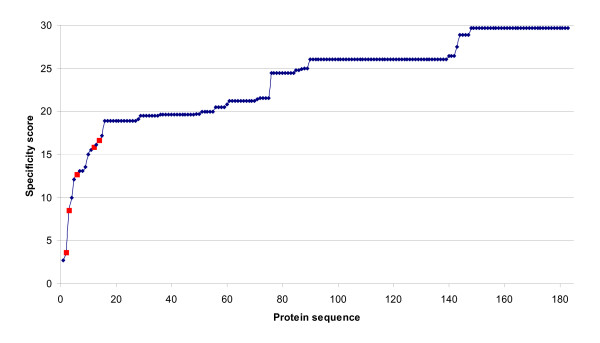
**Specificity scores for the dehydrogenase family**. The 183 LDH and MDH sequences are ordered according to specificity scores. The five wrongly assigned sequences are indicated in red.

Lactate (LDH) and malate (MDH) are subtypes of a large dehydrogenase family. They show considerable sequence variability [19] making them a more difficult case than the first two families. 183 dehydrogenase sequences (74 LDH and 109 MDH) were extracted from the UniProt database [17]. When the top 6 positions were used as a motif the dehydrogenases were split into an LDH and an MDH group with 5 wrong assignments (97% accuracy). The wrong assignments all had low specificity scores (Figure 1).

**Figure 2:**
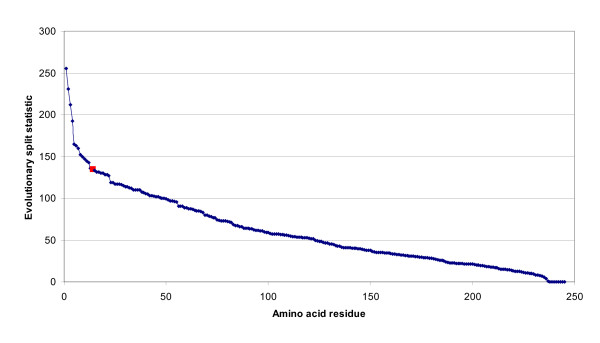
**Evolutionary split scores for amino acid residues of the dehydrogenase family**. The amino acid residues in the LDH/MDH multiple alignment are ordered using the evolutionary split score. Residue 144 of the alignment (Q in LDH, R in MDH) is shown in red.

The two residues with the highest evolutionary split scores were discussed by Hannenhalli and Russell [10] as important in determining the functional subtype. Experimentally it has been shown that a major determinant of the substrate specificity is the choice between glutamine or arginine at residue 144 (residue 102 of [19]). This position was the 14^th ^best evolutionary split score in our analysis (Figure 2). The reason why it does not rank higher is that arginine/glutamine exchanges are fairly common in proteins (and have a score of +1 in the BLOSUM-50 matrix used by the program).

The acyl transferase (AT) domains of Type I modular polyketide synthases (PKS) determine the substrate selection [4,5,20-23]. Most incorporate either a C2 unit (malonyl-CoA substrate) or a C3 unit (methylmalonyl-CoA substrate). The choice of substrate can be deduced from the chemical structure of the polyketide product. We chose 177 AT domains (99 C2, 78 C3). We used the top 7 positions to define a motif and the program divided the domains into C2 and C3 subtypes with only 5 wrong assignments (97% accuracy). The wrong assignments all had low specificity scores (among the lowest 6 scores of the 177 sequences). The top 7 amino acid positions chosen were positions previously recognized by Yadav and collaborators [9] by inspection of the sequences. The top 30 amino acid residues were identified in the sequence of *Escherichia coli *fatty acid synthase AT for which a 3-D protein structure has been determined ([24]; PDB ID 1MLA). The top 7 residues are in the region of the binding pocket where a direct effect on substrate binding might be expected. The other residues are scattered on the surface of the protein, too far from the substrate binding pocket to have a direct effect.

Ketoreductase domains (KR) of Type I modular PKSs use NADPH to stereospecifically reduce the initially formed keto group to a hydroxyl group [25]. The stereospecificity can only be deduced from the structure of the product for cases in which further reduction steps have not occurred. We used 72 KR domains for which the stereospecificity was known (33 R and 39 S). In this case, most of the residues with the best values for the evolutionary split parameter were clustered in a region of the sequence, so we chose the residues from positions 114 to 155 of the alignment to split the family into subtypes. This gave a 90% accurate assignment of domains (7 domains were misclassified). The motif residues included the residues that had been recognized by Caffrey [8] as playing a role in stereospecificity.

The final family that we examined was the small heat shock proteins (sHSP), where it is not clear whether there is a functional difference between different subtypes. We analysed 214 sequences and on the basis of the best four positions obtained a split between metazoan sHSPs and the others (plants, fungal, eubacterial and archaebacterial) (95% assignment) which corresponds to previously reported phylogenetic results [26]. The four residues (alignment positions 274, 292, 406 and 408) were localized on the 3-D structures of sHSPs from *Triticum aestivum *[27] and *Methanococcus janaschii *[28]. The four residues are in a region of the protein that is involved in dimerisation. It is known that oligomerisation is important for the function of the protein and this result suggests that the two subtypes identified might differ in oligomerisation properties.

The clustering algorithm allows a free choice of amino acids alignment positions to include in the motif. This raises the question as to how sensitive the clustering algorithm is to the exact choice of motif. We clustered the six protein families using amino acid positions with the highest evolutionary split scores and varying the length of the motifs from 5 to 30 positions. Table 2 shows that the accuracy of the clustering does not depend strongly on the number of positions chosen. This means that the algorithm could be used for the automatic clustering of protein families using a standard length of motif chosen from the best evolutionary split scores. In the case of the KR-domains, choosing a segment of the protein on the basis of specific knowledge, as done above, gave better results than using the best evolutionary split scores. The assignment of KR domains to subfamilies is complicated as they also determine the stereochemistry of methyl groups [29] and examination of 3-D structures of KR domains resulted in their division into six subtypes [30].

**Table 2:** Effect of motif length on clustering performance.

	**motif length**						
	**5**	**10**	**15**	**20**	**25**	**30**	

**Protein family**	**false assignments**						**No. sequences**

Nucleotidyl cyclases	0	0	0	0	0	0	75

Protein kinases	0	0	0	0	0	0	215

MDH/LDH	5	6	5	5	4	4	183

AT-domains	2	3	4	4	5	5	181

KR-domains	20	18	20	17	10	9	72

sHSP	10	13	14	11	5	5	214

The amino acids positions with the highest evolutionary split scores were used to construct the motifs.

The evolutionary split statistic allows the identification of residues that are important for the determination of subtypes. However, as it is calculated independently of the clustering, it is not as good as methods that are based on a known clustering. The subfamilies predicted by our clustering algorithm can be used for such analyses [10-14], which will give a more accurate identification of residues important for division into subtypes. The omission of sequences with low specificity scores should improve the analyses by removing misclassified sequences.

**Figure 3:**
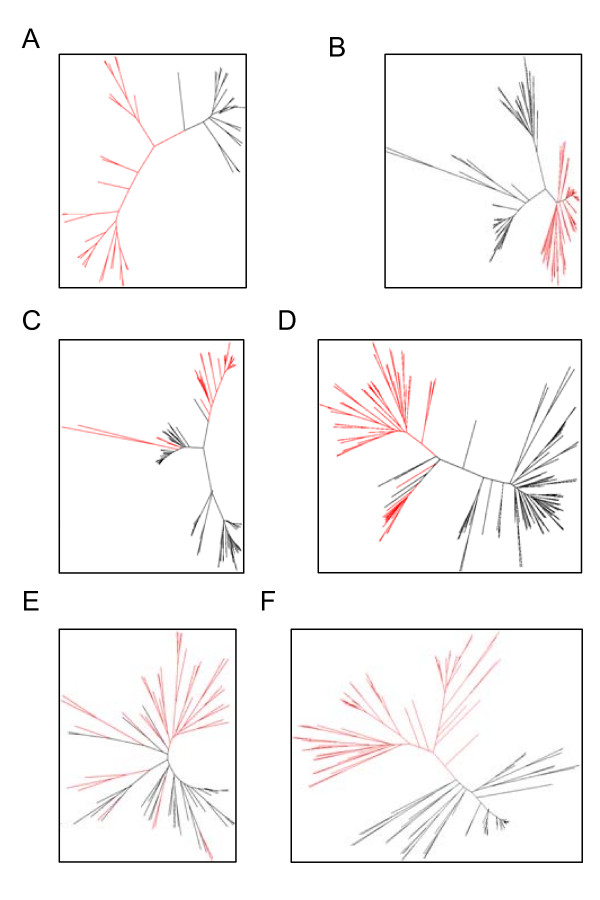
**Phylogenetic trees of the protein families**. The alignments of six protein families were used to construct phylogenetic trees from distances based on a BLOSUM matrix using a minimum evolution criterion. In each case, the branches corresponding to one of the two subfamilies are coloured red. (A) nucleotidyl cyclases (guanylate red), (B) protein kinases (tyrosine red), (C) dehydrogenases (LDH red), (D) AT-domains (C3 red), (E) KR-domains (S stereochemistry red), (F) sHSPs (metazooan black, others red).

The algorithm showed an efficient division into subtypes for the six protein families tested. An alternative approach to recognizing subtypes in the absence of functional information is to use phylogenetic analysis. In order to have a closer comparison with our clustering algorithm we constructed phylogenetic trees from the multiple alignments of our six protein families using distances calculated from a BLOSUM matrix [31] instead of the more common JTT method [32]. For the nucleotidyl cyclases and protein kinases, whose subtypes were recognised with complete accuracy by our method (Table 2), the functional subtypes do form separated clusters in the phylogenetic trees [Figure 3(A)) and 3(B)]. Division of the sequences into two subfamilies implies choosing a rooting point in the tree so that the subfamilies become clades in the rooted tree. In neither case, is the choice of such a rooting point unambiguous. For the cyclases [Figure 3(A))] there are several plausible rooting points, only one of which will give the correct subfamilies. The kinases [Figure 3(B))] fall into three clusters and the phylogenetic tree does not suggest the correct split into the two functional subtypes. The dehydrogenases [Figure 3(C))] also appear to split into three clusters and the phylogenetic tree does not suggest a division corresponding to the two functional subtypes, whereas our clustering program recognises the functional subtypes efficiently (Table 2). The AT-domains [Figure 3(D))] can be recognised as two groups using the phylogenetic tree with a similar degree of error to the clustering algorithm. The subtypes of the KR-domains [Figure 3(E))] cannot be recognised using the phylogenetic tree, whereas the two subtypes of the sHSPs are clear in the phylogenetic tree [Figure 3(F))]. Thus, in three of the six families, the phylogenetic trees did not give a clear identification of the functional subtypes. A further major advantage of the clustering algorithm is that the specificity score identifies sequences that are not well clustered by the algorithm so that they can be removed or treated with caution in subsequent analyses. The tests with known families showed that most wrong assignments involved such sequences.

In principle, the programs can also be used to cluster sequences into three or more subtypes. We tested for a clustering into three subtypes using two protein families: 92 serine proteases (67 trypsin-, 17 chymotrypsin-, 8 pancreatic elastase-subfamilies) and 59 AT-domains (28 incorporating methylmalonate, 18 malonate and 13 methoxymalonate). Clustering was undertaken using best 10, 20 and 30 positions for the evolutionary split statistic (data not shown). The clustering did not show a strong dependence on the number of positions. For the serine proteases, the trypsin subfamily was split into two groups and the chymotrypsin and pancreatic elastase subfamilies clustered together giving wrong clustering of 42 of the 92 sequences. Similarly, 22 of the 59 AT-domains were wrongly clustered. Thus, although the method works for carefully constructed sets of test data, it does not seem to be effective for real biological protein families. It is not surprising that the method becomes less effective with increasing number of subtypes. The potential of a column to contribute towards a *k*-way split is estimated with the evolutionary split statistic (formula 7) and increasing the number of subtypes drastically increases dimensionality of the parameter space; i.e. it is increasingly difficult to distinguish between evolutionary noise and functionally significant mutations. Thus, only exceedingly large sample sizes will provide sufficient power for the method to work well. Clustering is most efficient when the different subtypes are present in comparable numbers and the examples analysed in this paper show that the known sequences in natural protein families can often fall into one or two major subtypes with other subtypes being rare. Such situations can be analysed better by using binary clustering and subsequently looking for rarer subtypes in the sequences that have low specificity scores.

The method suffers from the drawback that it can only be used in practice for dividing protein families into two subtypes. This will cause problems for protein families with several common subtypes and the method may not work well for rare subtypes. Now that the feasibility of such a clustering algorithm has been demonstrated it is likely that improved algorithms can be devised to overcome these problems.

An important practical advantage of our algorithm is that it is computationally efficient allowing implementation on a public server. Using a standard PC with a 2 GHz processor, it needs about 0.1 second per column to compute the evolutionary split parameter (nearly independently of the number of sequences) and about 1 minute to compute the clustering into subtypes. It is therefore feasible to experiment with different motifs and different selections of the sequences to obtain optimal results. The method offers a useful tool to detect previously unsuspected clustering into subtypes. If experimental data for a limited number of proteins are available, they provide an independent test for the predicted clustering and the subtype of previously uncharacterised proteins is predicted.

## Conclusion

The programs cluster protein families into subtypes effectively without any prior functional knowledge. The specificity score identifies protein sequences that do not cluster well into the defined subtypes: these may include further rare subtypes. The programs are especially suitable for detecting novel unsuspected subtypes where extensive sequence data, but little experimental data are available.

## Methods

### Preparation of sequences

The amino acid sequences for 75 nucleotidyl cyclases, 183 dehydrogenases and 214 small heat shock proteins (sHSP) and 92 serine proteases were extracted from the UniProt database release 53.0 or 57.0 [33]. The amino acid sequences for 177 acyltransferase (AT) and 72 ketoreduction (KR) domains from modular polyketide synthases were obtained from the NRPS-PKS database [34,35]. The amino acid sequences of 85 serine/threonine and 130 tyrosine protein kinases were retrieved from the protein kinase database [18]. All 59 AT-domains were extracted from the following clusters: ascomycin, concanamycin, FK506, geldanamycin, herbimycin, niddamycin, soraphen using the MAPSI database [36]. Multiple alignments of the sequences were constructed using ClustalW and Clustal X [7,15,37]. These multiple alignments for each family are shown in additional materials.

### Construction of phylogenetic trees

Phylogenetic trees were constructed from the multiple alignments using the neighbour joining algorithm in version 3.66 of the PHYLIP package [38]. The distances were calculated with the Protdist program using the PMB (Probability Matrix from Blocks) model [31].

### A model of amino acid substitutions

Let A be the alphabet consisting of twenty standard amino acids, and let *q *= (*q*_1_, ..., *q*_20_) be the stationary (marginal) distribution of elements of A in some protein universe P. We denote by *e*_*i *_= (0,...,1,...0) the *i*-th vector in the canonical basis of R^*n*^, with 1 at the *i *-th position, and zeros elsewhere.

**Definition 1 **A *substitution model *for P is a family of distributions *a*_*i*, *t*_, *i *∈ *A*, *t *∈ [0, ∞ ⟩, that, for each *i *∈ *A*, satisfies

(1)

(2)

Here *q *= (*q*_*j*_) is the vector of frequencies with which amino acids occur in the family of proteins. For *a*_*i*, *t *_= (*a*_*i*, *t*_(1), ... *a*_*i*, *t*_(20)), *a*_*i*, *t*_(*j*) is, by definition, the probability of amino acid *i *mutating into *j *after time *t*; hence,

(3)

Let *A*_*t *_∈ *M*_20_(R) be defined by



so that *A*_*t *_is the matrix with vectors (*a*_*i*, *t*_)^*T *^as columns, for all *t*. If we assume that *A*_*t*_, in addition to (1) and (2), satisfies

(4)

then *A*_*t *_is the matrix of transition probabilities of a homogenous Markov process and can be written as

(5)

describing the evolution of elements of A within the class P. There are several examples of such models in the context of biological sequence analysis, most notably the PAM series of matrices [39] - in the case of amino acid evolution - and Jukes-Cantor or Kimura matrices [40] in the case of DNA evolution. Now, we will present a simple substitution model, based on the BLOSUM matrices [16] - or, for that matter, on any substitution matrix - which does not necessarily arise from an evolutionary Markov process, but suffices for our purposes.

It is well known that the BLOSUM50 matrix is defined by , where *p*_*i*, *j *_indicates the probability of seeing amino acids *i *and *j *substitute each other in a homologous sequence. This matrix can also be written as

(6)

for *s *= log_*e*_2. Varying *s *in the above equation will, after renormalisation and reparametrisation *t *= *s*^-1 ^yield a family *a*_*i*, *t *_as above. This way of obtaining transition probabilities is clearly different and simpler than (5). However, it will produce a rich class of probability distributions that reflect relations between amino acids captured by BLOSUM scores.

### Calculation of the evolutionary split statistic

In this section, we describe the *evolutionary split *(es) statistic. It will be used to predict positions in the multiple alignment that are potentially significant for functional clustering.

**Definition 2 **Let *D *denote a column in a multiple alignment, and assume that *D *contains no gaps. Then

(7)

where *b*, *b*_*i *_are substitution distributions from Definition 1, , with λ_*i*_≥ 0 and *k *is the number of subtypes that we are searching for. The algorithm was implemented as a C program.

**Remark **Note that *es*_*k*_(·) compares the likelihood of the data with respect to the optimal mixture of *k *substitution models, with the likelihood under a single optimal model. In practice, we used a discrete approximation of the parameter space for the optimization. Also, a mild sequence weighting scheme was applied, to correct for the lack of independence in the sample (see [41]).

### Clustering algorithm

Let us suppose that *l *columns (with no gaps) have been selected from the multiple alignment. Hence, we are dealing with *n *protein sequences *y *= {*y*^1^, ..., *y*^*n*^}, all of the same length *l*, i.e , for all *i*. We want to define a model for dividing *y *into *k *subsets. Let *I *= (*I*_1_, ..., *I*_*k*_) stand for a partition of {1, ..., *n*} into *k *non-empty disjoint subset. A model for our data set *y *= {*y*^1^, ..., *y*^*n*^} consists of two components -- a partition *I *= (*I*_1_, ..., *I*_*k*_) and the parametric model *M *itself, which consists of *k *sequences of distributions from the substitution model, e.g. , for *j *= 1, ..., *k*. We obtain the clustering by optimizing the following expression

(8)

where

(9)

Thus, we rely on the conditional likelihood to cluster our data in *k *groups. By doing so, we effectively treat the partition *I *= (*I*_1_, ..., *I*_*k*_) as a (discrete) parameter in the model.

A more traditional approach is to consider the real likelihood of the data with respect to the mixture model, and treat the membership of the clusters as missing data. In such a framework, the model *M *consists of parameters λ_*i *_∈ [0,1), with  and *k *sequences of distributions from the substitution model as above. The model for the data is obtained by maximization of the log-likelihood



where

(10)

Given the optimal model *M *= {(λ_*i*_, *M*_*i*_)}, we can obtain the clustering using the following Bayesian criterion

(11)

Clearly the expression we need to optimize if we choose the conditional likelihood is much simpler, although the parameter space is somewhat more complicated. In either case, finding the optimal model is a difficult problem. For real-life data sets, the clustering will not differ if we choose one approach or the other, but the conditional likelihood procedure tends to reach the optimum much faster than the standard deterministic annealing EM-algorithm [42]. In some applications it might be more reasonable to take fully Bayesian approach and report posterior probabilities for each clustering obtained. However, our aim in the present paper was to obtain one useful partition of data sets and we did not explore this point of view further. In the rest of this section we describe a natural optimization method for the conditional likelihood approach.

Let us now describe the optimization algorithm. A clustering of the data set *y *= {*y*^1^, ..., *y*^*n*^} will be denoted by *I *= (*I*_1_, ..., *I*_*k*_) -- same as the associated partition, and let *M*_*i*_, *i *= 1, ..., *n *denote the (parametric) model corresponding to the *i*-th cluster. As already mentioned, the following algorithm is a natural solution:

•Step1: choose an initial clustering (*I*_1_, ..., *I*_*k*_)

•Step2: determine the optimal model *M*_*i *_for the *i*-th cluster, for all *i*

•Step3: for each *y*^*j*^, change cluster membership by setting *y*^*j *^∈ *I*_*l *_if and only if *P*(*y*^*j*^| *M*_*l*_) ≥ *P*(*y*^*j*^| *M*_*i*_), for all *i*

•Step4: goto Step2

It is easy to show that this procedure increases the value of the likelihood function from (9), so will always reach a (local) maximum (if a sufficient number of iterations has been performed). In order to avoid local maxima, we use *smoothing*, i.e. we use the uniform distribution  to obtain modified model  as a convex combination of *M*_*j *_and *u *in Step2. Clearly, the amount of smoothing should be reduced as the optimization process progresses. Furthermore, we use simulated--annealing like acceptance-rejection principle for the cluster membership: the proposal in the Step3 is accepted with probability



where *T *is the temperature, + ∞ → *T *→ 0. So, with these additions, we get the following algorithm:

•Step1: choose an initial clustering (*I*_1_, ..., *I*_*k*_)

•Step2: determine the optimal model *M*_*i *_for the *i*-th cluster, for all *i*

•Step2': *M*_*i *_is replaced with , for all *i*

•Step3': for each *y*^*j*^, propose cluster membership change by setting *y*^*j *^∈ *I*_*l *_if and only if *P*(*y*^*j*^| *M*_*l*_) ≥ *P*(*y*^*j*^| *M*_*i*_), for all *i*, and accepting it with probability ; if proposal is rejected, the cluster membership is assigned randomly

•Step4: goto Step2

The algorithm was implemented as a C program.

## Availability

The programs are offered on a web server at: . Further details of the programs can be obtained from PG.

## Authors' contributions

PG developed the mathematical background of the clustering concept, produced the programs and wrote the initial draft of the manuscript. BB developed the statistical ideas. JZ, DV, AK and CE carried out the analyses of the protein families. DH, PFL and JC contributed biological ideas to the development of the methodology and drafted the final manuscript. All authors read and approved the final manuscript.

## Supplementary Material

Additional file 1 **Alignments**. The alignments used to test the clustering method: nucleotidyl cyclases, protein kinases, dehydrogenases, acyl transferases, ketoreductases and small heat shock proteins. Click here for file

Additional file 2 **Detailed output of the evolutionary split and clustering programs**. For each of the protein families (nucleotidyl cyclases, protein kinases, dehydrogenases, acyl transferases, ketoreductases and small heat shock proteins) there are two tables. The evolutionary split table has the following columns: residue position in the alignment (only residues that are present in every protein sequence are used for the calculation), amino acid for one ancestor model, log likelihood for one ancestor model, amino acids for two ancestor model, log likelihood for two ancestor model, evolutionary split statistic. The clustering table has the following columns: name of protein sequence, log likelihood for membership of subtype **a**, log likelihood for membership of subtype **b**, predicted subtype, specificity score. Click here for file

## References

[B1] Altschul SF, Gish W, Miller W, Myers EW, Lipman DJ (1990). Basic local alignment search tool. J Mol Biol.

[B2] Eddy SR (1998). Profile hidden Markov models. Bioinformatics.

[B3] Bateman A, Birney E, Cerruti L, Durbin R, Etwiller L, Eddy SR, Griffiths-Jones S, Howe KL, Marshall M, Sonnhammer EL (2002). The Pfam protein families database. Nucleic Acids Res.

[B4] Hranueli D, Cullum J, Basrak B, Goldstein P, Long PF (2005). Plasticity of the *Streptomyces *genome - evolution and engineering of new antibiotics. Curr Med Chem.

[B5] Chan YA, Podevels AM, Kevany BM, Thomas MG (2009). Biosynthesis of polyketide synthase extender units. Nat Prod Rep.

[B6] Starcevic A, Zucko J, Simunkovic J, Long PF, Cullum J, Hranueli D (2008). *ClustScan*: An integrated program package for the semi-automatic annotation of modular biosynthetic gene clusters and *in silico *prediction of novel chemical structures. Nucleic Acids Res.

[B7] Thompson JD, Higgins DG, Gibson TJ (1994). CLUSTAL W: improving the sensitivity of progressive multiple sequence alignment through sequence weighting, position-specific gap penalties and weight matrix choice. Nucleic Acids Res.

[B8] Caffrey P (2003). Conserved amino acid residues correlating with ketoreductase stereospecificity in modular polyketide synthases. Chem Bio Chem.

[B9] Yadav G, Gokhale RS, Mohanty D (2003). Computational approach for prediction of domain organization and substrate specificity of modular polyketide synthases. J Mol Biol.

[B10] Hannenhalli SS, Russell RB (2000). Analysis and prediction of functional sub-types from protein sequence alignments. J Mol Biol.

[B11] Pirovano W, Feenstra KA, Heringa J (2006). Sequence comparison by sequence harmony identifies subtype-specific functional sites. Nucleic Acids Res.

[B12] Pazos F, Rausell A, Valencia A (2006). Phylogeny-independent detection of functional residues. Bioinformatics.

[B13] Wallace IM, Higgins DG (2007). Supervised multivariate analysis of sequence groups to identify specificity determining residues. BMC Bioinformatics.

[B14] Ye KK, Feenstra A, Heringa J, IJzerman AP, Marchiori E (2008). Multi-RELIEF: a method to recognize specificity determining residues from multiple sequence alignments using a machine-learning approach for feature weighting. Bioinformatics.

[B15] Thompson JD, Gibson TJ, Plewniak F, Jeanmougin F, Higgins DG (1997). The CLUSTAL X windows interface: flexible strategies for multiple sequence alignment aided by quality analysis tools. Nucleic Acids Res.

[B16] Henikoff S, Henikoff JG (1992). Amino acid substitution matrices from protein blocks. Proc Natl Acad Sci USA.

[B17] The UniProt Consortium (2009). The Universal Protein Resource (UniProt) 2009. Nucleic Acids Res.

[B18] Smith CM, Shindyalov IN, Veretnik S, Gribskov M, Taylor SS, Ten Eyck LF, Bourne PE (1997). The protein kinase resource. Trends Biochem Sci.

[B19] Wilks HM, Hart KW, Feeney R, Dunn CR, Muirhead H, Chia WN, Barstow DA, Atkinson T, Clarke AR, Holbrook JJ (1988). A specific, highly acitve malate dehydrogenase by redesign of a lactate dehydrogenase framework. Science.

[B20] Haydock SF, Aparicio JF, Molnár I, Schwecke T, Khaw LE, König A, Marsden AF, Galloway IS, Staunton J, Leadlay PF (1995). Divergent sequence motifs correlated with the substrate specificity of (methyl)malonyl-CoA:acyl carrier protein transacylase domains in modular polyketide synthases. FEBS Lett.

[B21] Lau J, Fu H, Cane DE, Khosla C (1999). Dissecting the role of acyltransferase domains of modular polyketide synthases in the choice and stereochemical fate of extender units. Biochemistry.

[B22] Reeves CD, Murli S, Ashley GW, Piagentini M, Hutchinson CR, McDaniel R (2001). Alteration of the substrate specificity of a modular polyketide synthase acyltransferase domain through site-specific mutations. Biochemistry.

[B23] Del Vecchio F, Petkovic H, Kendrew SG, Low L, Wilkinson B, Lill R, Cortés J, Rudd BA, Staunton J, Leadlay PF (2003). Active-site residue, domain and module swaps in modular polyketide synthases. J Ind Microbiol Biotechnol.

[B24] Serre L, Verbree EC, Dauter Z, Stuitje AR, Derewenda ZS (1995). The *Escherichia coli *malonyl-CoA:acyl carrier protein transacylase at 1.5A resolution. Crystal structure of a FAS component. J Biol Chem.

[B25] Castonguay R, He W, Chen AY, Khosla C, Cane DE (2007). Stereospecificity of ketoreductase domains of the 6-deoxyerythronolide B synthase. J Am Chem Soc.

[B26] Waters ER, Lee GJ, Vierling E (1996). Evolution, structure and function of the small heat shock proteins in plants. J Exp Bot.

[B27] van Montfort RL, Basha E, Friedrich KL, Slingsby C, Vierling E (2001). Crystal structure and assembly of a eukaryotic small heat shock protein. Nat Struct Biol.

[B28] Kim KK, Kim R, Kim SH (1998). Crystal structure of a small heat-shock protein. Nature.

[B29] Starcevic A, Jaspars M, Cullum J, Hranueli D, Long PF (2007). Predicting the nature and timing of epimerisation on a modular polyketide synthase. Chem Bio Chem.

[B30] Keatinge-Clay AT (2007). A tylosin ketoreductase reveals how chirality is determined in polyketides. Chemistry & Biology.

[B31] Veerassamy S, Smith A, Tillier ERM (2003). A transition probability model for amino acid substitutions from blocks. J Comput Biol.

[B32] Jones DT, Taylor WR, Thornton JM (1992). The rapid generation of mutation data matrices from protein sequences. Comput Appl Biosci.

[B33] ExPASy Proteomics Server. http://expasy.org/.

[B34] NRPS_PKS: A knowledge based resource for analysis of Non-ribosomal Peptide Synthetases and Polyketide Synthases. http://www.nii.res.in/nrps-pks.html.

[B35] Ansari MZ, Yadav G, Gokhale RS, Mohanty D (2004). NRPS-PKS: a knowledge-based resource for analysis of NRPS/PKS megasynthases. Nucleic Acids Res.

[B36] Tae H, Jae KS, Park K (2009). Development of an analysis program of Type I polyketide synthase gene clusters using homology search and profile hidden Markov model. J Microbiol Biotechnol.

[B37] European Bioinformatics Institute. http://www.ebi.ac.uk.

[B38] Felsenstein J (1989). PHYLIP - Phylogeny Inference Package (Version 3.2). Cladistics.

[B39] Dayhoff MO, Schwartz RM, Orcutt BC (1978). A model of evolutionary change in proteins. Atlas of Protein Sequence and Structure.

[B40] Felsenstein J (2004). Inferring Phylogenies.

[B41] Henikoff S, Henikoff JG (1994). Position-based sequence weights. J Mol Biol.

[B42] Ueda N, Nakano R (1998). Deterministic Annealing EM Algorithm. Neural Networks.

